# Herpes Simplex Virus Type 1 Hepatitis in an Immunocompetent Female After Laparoscopic Cholecystectomy

**DOI:** 10.7759/cureus.9529

**Published:** 2020-08-03

**Authors:** Dallis Q Ngo, Christina Vu, Xolani Mdluli, Katrina Platt, Mehrdad Asgeri

**Affiliations:** 1 Pulmonary Medicine, Saint Peter's University Hospital, New Brunswick, USA; 2 Infectious Diseases, University of Southern California, Los Angeles, USA; 3 Infectious Diseases, Desert Regional Medical Center, Palm Springs, USA; 4 Internal Medicine, Desert Regional Medical Center, Palm Springs, USA; 5 Gastroenterology, Desert Regional Medical Center, Palm Springs, USA

**Keywords:** hsv, hsv-1, herpes simplex virus type 1, hepatitis

## Abstract

We present a rare case of a healthy, non-pregnant, middle-aged and immunocompetent woman who underwent laparoscopic cholecystectomy for acute cholecystitis with a post-operative course complicated by herpes simplex virus type 1 (HSV-1) hepatitis secondary to post-surgical inflammation. Her initial post-operative course was complicated by intermittent fevers, leukocytosis, jaundice, elevated transaminases, and right upper quadrant abdominal pain, and she was subsequently placed on broad-spectrum antibiotics with no improvement. During her hospital course, the patient developed herpes labialis, and HSV-1 hepatitis was confirmed by serology and HSV-1 polymerase chain reaction (PCR), in lieu of a liver biopsy. After this was discovered, the patient was placed on valacyclovir and had a successful response. The importance of this case is to emphasize the possibility of herpes simplex virus (HSV) hepatitis as a post-operative complication and the benefit of early empiric antiviral treatment.

## Introduction

Herpes simplex virus type 1 (HSV-1) is a highly prevalent mucocutaneous infection worldwide, however herpes simplex virus (HSV) hepatitis is a rare complication with high morbidity and mortality, accounting for 0.8% of all acute liver failure cases [1,2]. Many cases of HSV hepatitis have been previously reported in the literature in pregnant women and individuals who are immunosuppressed for treatment of various medical conditions such as malignancies. We present an exceedingly rare case of a healthy immunocompetent woman, status post-laparoscopic cholecystectomy, with a post-operative course complicated by HSV hepatitis with a favorable outcome.

## Case presentation

**Figure 1:**
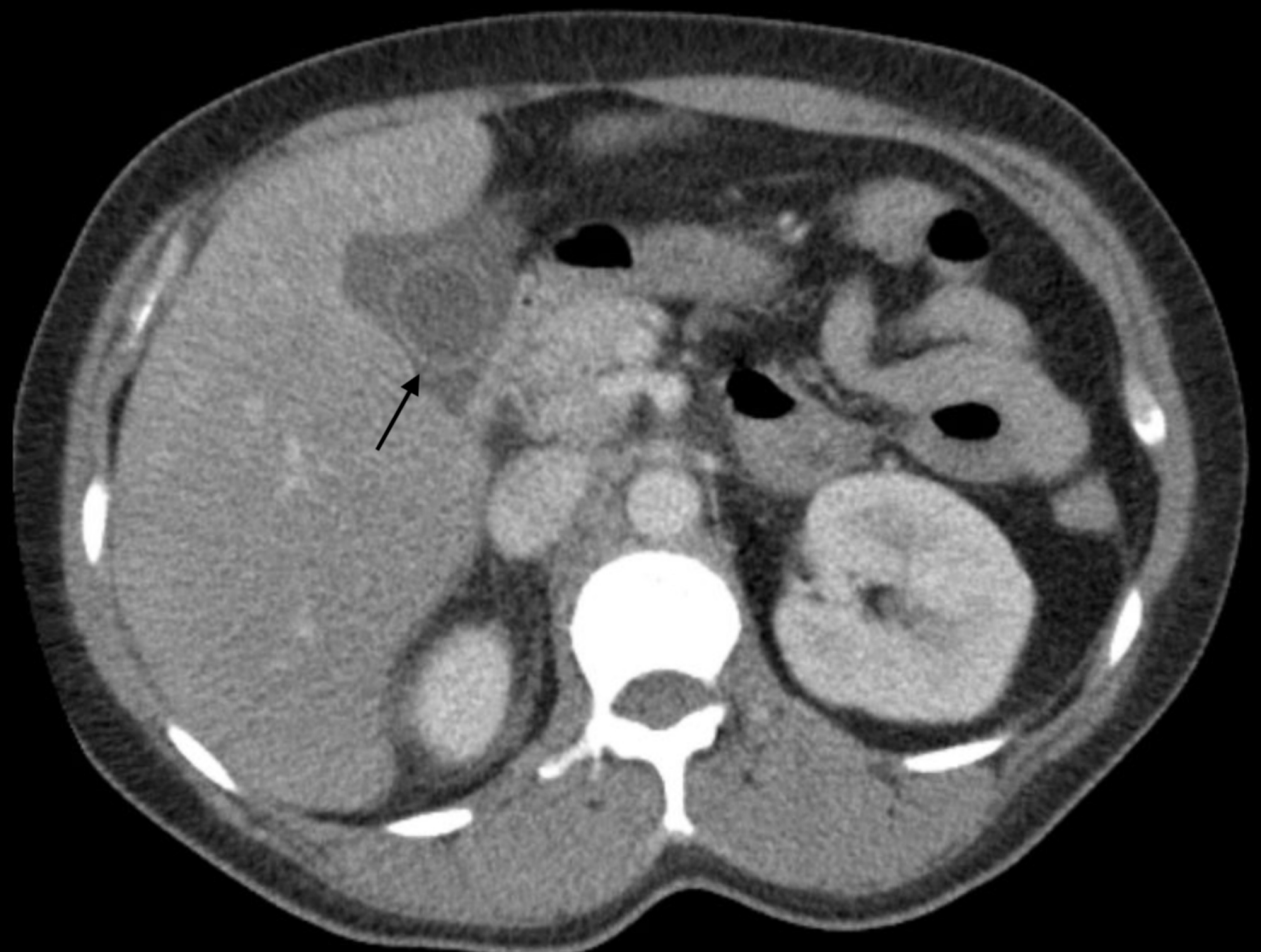
CT Abdomen & Pelvis with Contrast - Before Laparoscopic Cholecystectomy Black arrow: Gallbladder with pericholecystic fluid

A 48-year-old Hispanic female with no prior medical history, including sexually transmitted infections, presented as a transfer from an outside hospital for higher level of care due to concerns of sepsis possibly secondary to ascending cholangitis versus bile leak. The patient initially presented to the outside hospital with a diagnosis of acute cholecystitis and underwent a laparoscopic cholecystectomy (Figure 1). The tissue pathology confirmed evidence of acute cholecystitis. Her post-operative course was complicated by intermittent fever and tachycardia followed by complaints of 5/10 diffuse abdominal pain without abdominal rebound or guarding by day four. She appeared fatigued with minimal scleral icterus. Her laparoscopic wounds showed no signs of active infection. Chest x-ray did not demonstrate acute airspace disease. Hepatobiliary iminodiacetic acid (HIDA) scan demonstrated surgically absent gallbladder, homogenous uptake throughout the liver and no bile leak. CT of the abdomen and pelvis with contrast demonstrated free fluid adjacent to the right hepatic lobe, likely post-operative free fluid, however no abscess, pancreatitis, or hepatic lesions appreciated. She was started initially on piperacillin-tazobactam and metronidazole antibiotics at the outside hospital without clinical improvement, then switched to vancomycin and meropenem once transferred to the intensive care unit (ICU) at our facility.

**Figure 2:**
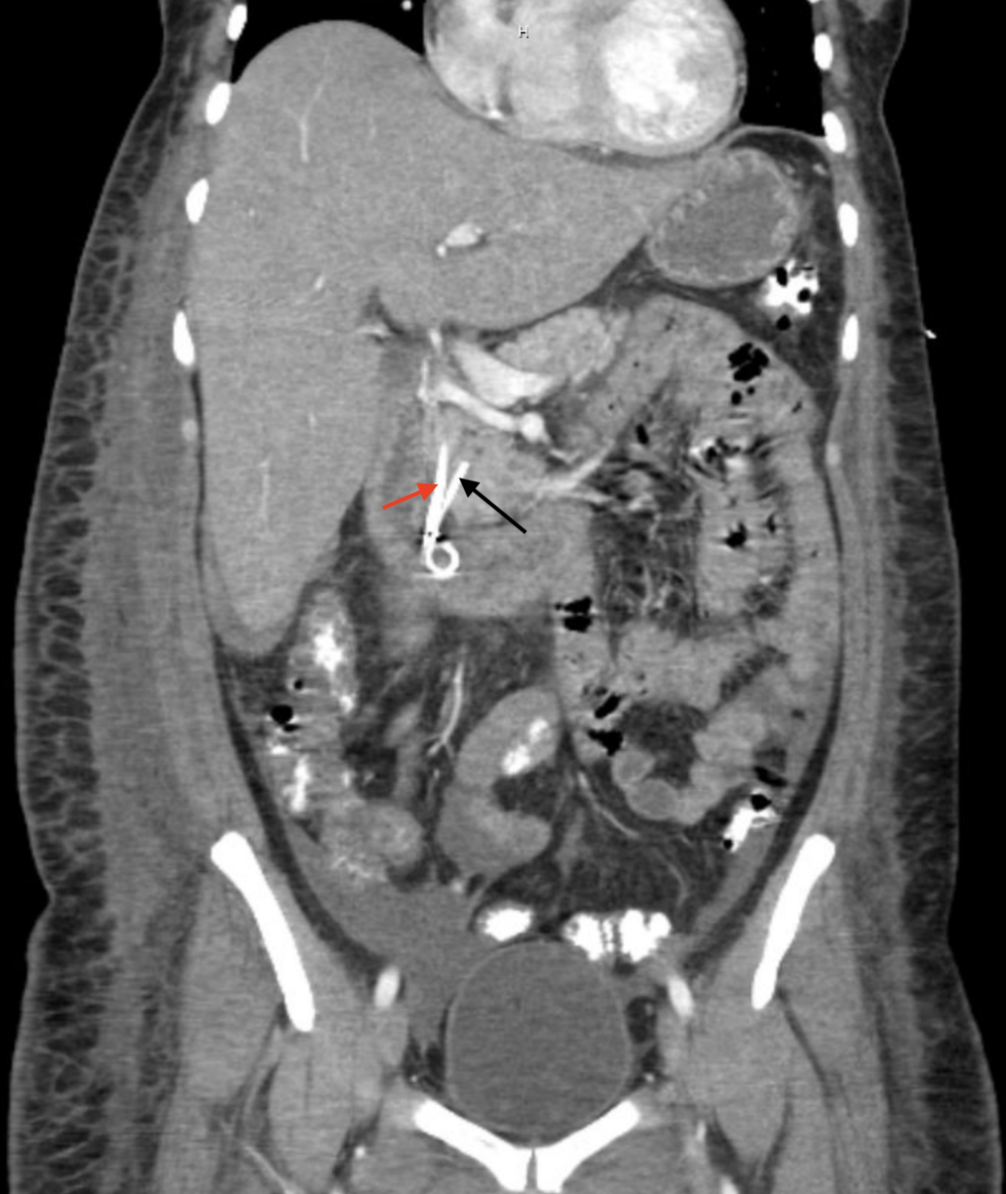
CT Abdomen & Pelvis with Contrast - Common Bile Duct & Pancreatic Duct Stents Black arrow: 4-French 3-cm pancreatic duct stent. Red arrow: 8.5-French 9-cm common bile duct stent.

**Figure 3:**
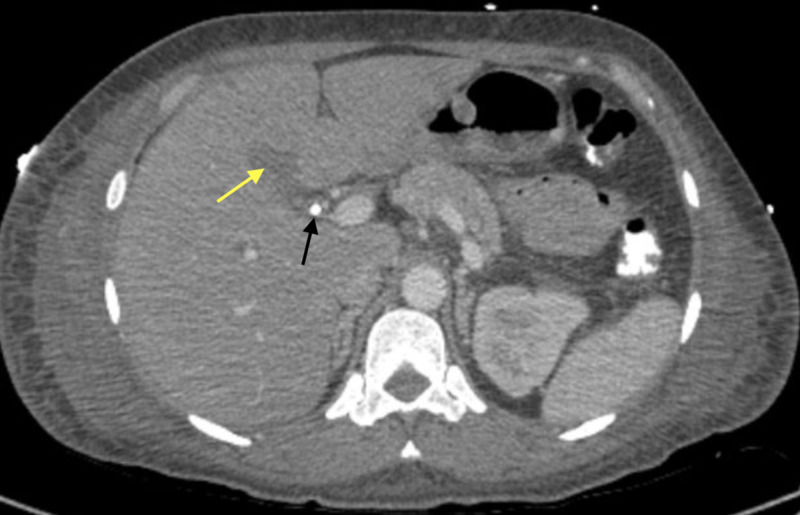
CT Abdomen & Pelvis with Contrast - After Laparoscopic Cholecystectomy & Stent Placement Black arrow: Common bile duct stent Yellow arrow: Decreased fluid collection in the gallbladder fossa

On presentation to our hospital, she was febrile at 39.2°C. Laboratory studies showed a leukocytosis of 18.4 x 10^9^ white blood cells (WBC)/L, hemoglobin (Hgb) 7 g/dL, platelets 57x10^9^/L, international normalized ratio (INR) 1.1, albumin 2.3 g/dL, alkaline phosphatase (ALP) 229 units/L (upper limit of normal [ULN] 126 units/L), alanine aminotransferase (ALT) 104 units/L (ULN 50 units/L), aspartate aminotransferase (AST) 67 units/L (ULN 36 units/L), and total bilirubin 1.9 mg/dL (ULN 1.3 mg/dL). D-dimer and fibrinogen were elevated at 6.89 mg/L (ULN 0.59 mg/L) and 445 mg/L (ULN 430 mg/L), respectively. Her viral and autoimmune hepatitis panels were negative. She underwent an endoscopic retrograde cholangiopancreatography (ERCP) which did not demonstrate a bile leak, although sludge and gravel were present. Therefore, an 8.5-French 9-cm stent was placed in the distal common bile duct (CBD) and a 4-French 3-cm single pigtail pancreatic stent was placed to protect against pancreatitis (Figure 2). Over the next eight days, she continued to have multiple elevated body temperatures of >38°C, her leukocytosis did not improve and her transaminases remained persistently elevated. Repeat CT of the abdomen and pelvis with contrast showed an improvement of the gallbladder fossa fluid collection (Figure 3). Her blood, urine, and sputum cultures remained negative. The patient started to complain of oral pain to which numerous ulcerated lesions are appreciated on her anterior tongue, soft palate, and lower right lip, which were of presumed HSV infection previously not present on admission. Valacyclovir 1g PO every eight hours (q8h) was started for empiric treatment. Her serologies were highly positive for HSV-1 IgG 46.8 (reference range [RR] negative: <0.91 index) and HSV-1/2 IgM ratio 2.59 (RR positive: >1.09 ratio). Confirmatory HSV-1 DNA polymerase chain reaction (PCR) was positive and negative for HSV-2 and HIV. The patient remained afebrile for two days afterward, during which her leukocytosis improved to 7.7 x 10^9^ WBC/L, liver function test improved with ALP 121 units/L, total bilirubin 0.8 mg/dL, and AST and ALT 43 units/L and 39 units/L, respectively. She was discharged from the hospital with a continued course of valacyclovir. Her outpatient follow-up revealed resolution of oral lesions, abdominal symptoms, and abnormal transaminases.

## Discussion

Based on population data in 2012, herpes simplex virus type 1 (HSV-1) is a highly prevalent mucocutaneous infection among individuals aged 0 - 49 with an estimated average worldwide prevalence of 67%. The highest prevalence is found in Africa at 87% and the lowest in the Americas at 40-50% [1]. Disseminated HSV hepatitis, however, is a rare complication and potentially fatal disorder that accounts for less than 2% of all viral etiologies and only 0.8% of all acute liver failure cases [2]. The majority of HSV hepatitis cases reported in current literature involve pregnant patients or those who are immunocompromised as a result of immunosuppressant treatment of malignancies or status post organ transplantation [2].

HSV hepatitis as a post-surgical complication in a healthy immunocompetent patient, as in our case, is considered exceedingly rare [3]. In a 15-case review by Yokoi et al., various types of surgeries were involved, however, no specific procedure has been demonstrated to suggest any relationship to HSV hepatitis [3]. The pathogenesis of HSV hepatitis is unclear at the moment but there is concern that surgical manipulation may cause excessive inflammation resulting in immune modulation. This source of physiological stress and inflammation may lend itself to a unique post-surgical course characterized by reactivation of previously dormant HSV-1 infection, HSV-1 viremia, and subsequent acute liver injury or failure secondary to HSV hepatitis [3].

We cannot exclude the possibility that our patient had HSV hepatitis on initial presentation prior to her laparoscopic cholecystectomy. It is not standard practice to routinely screen patients for HSV hepatitis when they present with complaint of right upper quadrant abdominal pain and elevated transaminases suggestive of hepatocellular damage. However, it is important to remember to keep the diagnosis within the list of differential diagnoses. Early empiric treatment would have mitigated her risk of progression to liver failure, shortened her prolonged hospitalization, and saved the healthcare system hundreds of thousands of dollars.

Our patient was previously infected with HSV-1 as demonstrated by her positive IgG serology, though at the time of surgery, she was not demonstrating clinical signs of HSV-1 reactivation. During her post-surgical course, however, she demonstrated nonspecific signs of HSV-1 reactivation and hepatitis as characterized by fever, abdominal pain, sharply elevated transaminases, and slight elevation in total bilirubin [4]. This was mistaken for more common diagnoses given the clinical scenario such as ascending cholangitis, peritonitis, or bile leak and thus treated as such, albeit unsuccessfully. By day eight, her herpes labialis became apparent, resulting in appropriate empiric treatment with valacyclovir 1g orally every eight hours and confirmation with positive HSV-1 IgM and HSV-1 PCR. Similar to our patient’s onset of symptoms, Yokoi et al. demonstrated that with HSV hepatitis, high fever is the universal first sign with median onset by day 4.5, followed by gastrointestinal symptoms on day seven and liver dysfunction by day 8.5 [3]. Mucocutaneous lesions such as herpes labialis are a classic sign commonly seen with HSV-1 reactivation yet its absence does not rule out HSV as cause for hepatitis due to it being reported in less than 50% of HSV hepatitis patients [5,6]. Coagulopathy and thrombocytopenia may be observed [5,7].

The gold standard diagnostic test for HSV hepatitis is histopathologic evidence by liver biopsy which would demonstrate hepatic necrosis, intranuclear inclusions, and positive immunohistochemical staining using labeled HSV specific monoclonal antibodies [8]. Because of the invasive nature and risks of further complications such as hemorrhage in unstable patients, such as our case, liver biopsy may not be a reasonable option. The accuracy of HSV IgM serology for diagnosis of HSV hepatitis has also come into question as demonstrated in a study by Levitsky et al. where 11% of their patient population had a positive HSV-1/2 IgM titer of 1:20 despite a negative high sensitivity qualitative PCR assay, representing false positivity [9]. As such, the utility of HSV PCR for confirmatory diagnosis of HSV hepatitis has been suggested as a method for an accurate, rapid, and non-invasive diagnosis versus inaccurate and invasive tests such as serology and liver biopsy [9]. Empiric treatment with parenteral acyclovir 5-10 mg/kg every eight hours may reverse the disease process if instituted early and is recommended for patients who present with acute liver failure of unknown etiology until HSV hepatitis is excluded [10]. Our case involved acute liver injury therefore we believed oral valacyclovir at a dose of 1g every eight hours was appropriate. If left untreated, patients may progress to fulminant liver failure and disseminated intravascular coagulation with high rates of morbidity and mortality, therefore early therapy is paramount and essential [6]. 

## Conclusions

Herpes simplex virus type 1 (HSV-1) hepatitis as a post-operative complication in an immunocompetent female is an exceedingly rare occurrence. However, high clinical suspicion, early detection, and treatment is essential for good patient outcomes with this disease. Our complicated case provides an example of the clinical utility of HSV PCR as an alternative method for confirmatory diagnosis of HSV hepatitis in lieu of a liver biopsy.

## References

[1] Looker KJ, Magaret AS, May MT, Turner KM, Vickerman P, Gottlieb SL, Newman LM (2015). Global and regional estimates of prevalent and incident herpes simplex virus type 1 infections in 2012. PLoS One.

[2] Down C, Mehta A, Salama G (2016). Herpes simplex virus hepatitis in an immunocompetent host resembling hepatic pyogenic abscesses. Case Reports Hepatol.

[3] Yokoi Y, Kaneko T, Sawayanagi T, Takano Y, Watahiki Y (2018). Fatal fulminant herpes simplex hepatitis following surgery in an adult. World J Clin Cases.

[4] Berrington WR, Jerome KR, Cook L, Wald A, Corey L, Casper C (2009). Clinical correlates of herpes simplex virus viremia among hospitalized adults. Clin Infect Dis.

[5] Allen RH, Tuomala RE (2005). Herpes simplex virus hepatitis causing acute liver dysfunction and thrombocytopenia in pregnancy. Obstet Gynecol.

[6] Arkin LM, Castelo-Soccio L, Kovarik C (2009). Disseminated herpes simplex virus (HSV) hepatitis diagnosed by dermatology evaluation. Int J Dermatol.

[7] Flewett TH, Parker RG, Philip WM (1969). Acute hepatitis due to herpes simplex virus in an adult. J Clin Pathol.

[8] Phadke VK, Friedman-Moraco RJ, Quigley BC, Farris AB, Norvell JP (2016). Concomitant herpes simplex virus colitis and hepatitis in a man with ulcerative colitis. Medicine.

[9] Levitsky J, Duddempudi AT, Lakeman FD (2008). Detection and diagnosis of herpes simplex virus infection in adults with acute liver failure. Liver Transpl.

[10] Norvell JP, Blei AT, Jovanovic BD, Levitsky J (2007). Herpes simplex virus hepatitis: an analysis of the published literature and institutional cases. Liver Transpl.

